# Heterogeneous Microstructure and Tensile Properties of Fe_50_Mn_30_Co_10_Cr_10_ Metastable High-Entropy Alloy

**DOI:** 10.3390/ma17235893

**Published:** 2024-12-01

**Authors:** Xiuying Sun, Wei Zhou, Zhanjiang Li, Chunfu Hong, Fa Chang, Jun Tian, Pinqiang Dai

**Affiliations:** 1 College of Materials Science and Engineering, Fujian University of Technology, Fuzhou 350118, China; 2221602014@smail.fjut.edu.cn (X.S.); 2221604051@smail.fjut.edu.cn (W.Z.); c.f.hong@fjut.edu.cn (C.H.); cfzyzs_2011@163.com (F.C.); tianj@fjut.edu.cn (J.T.); 2 Fujian Provincial Key Laboratory of New Material Preparation and Forming Technology, Fuzhou 350118, China; 3 School of Naval Architecture & Intelligent Manufacturing, Jiangsu Maritime Institute, Nanjing 211170, China; l6z6j6@126.com

**Keywords:** metastable high-entropy alloys, Fe_50_Mn_30_Co_10_Cr_10_, heterogeneous microstructure, strength and ductility, mechanical properties

## Abstract

Face-centered cubic (FCC)-structured high-entropy alloys (HEAs) are facing a major challenge due to a trade-off between strength and ductility. In this paper, we systematically investigated the microstructural evolution and tensile properties of metastable dual-phase (DP) FeMnCoCr HEAs via cold rolling and partial recrystallized annealing, which resulted in a heterogeneous microstructure, and by inducing strengthening and strain-hardening through heterogeneous deformation-induced (HDI) strategies. The results show that the alloy was annealed at 600 °C for 10 min, exhibiting a good combination of strength and ductility. A higher alloy strength was obtained via HDI strengthening, while FCC → HCP phase transformation, deformation twins, and HDI strain-hardening contributed to the excellent ductility. The results provide a viable method for the design of subsequent alloys.

## 1. Introduction

High-entropy alloys (HEAs) were first proposed by Yeh [1] et al. in 2004. Pentaelemental-and-above equiatomic HEAs were initially developed for research purposes. With more in-depth research, the definition of HEAs has been revised. Now, alloys composed of four elements with near equimolar ratios can also be considered as HEAs [2,3,4]. Despite the highly complex chemical composition, the phase constitution of HEAs is often simple, usually with a single-phase or dual-phase structure. HEAs exhibit four major effects, including severe lattice distortion, slow diffusion, cocktail, and high entropy effect [5]. Meanwhile, due to unique mechanical properties, HEAs greatly expand the range of compositional design in metallic material and are considered to be a promising material [6,7]. According to the report [8], the global high-entropy alloys market was valued at USD 54.7 million in 2022, and the market is expected to reach USD 486.3 million by 2032, growing at a compound annual growth rate of 24.42% during the forecast period, which demonstrates its importance in the field of materials and has attracted a lot of attention, prompting an in-depth study in this paper.

Face-centered cubic (FCC)-structured HEAs have attracted a lot of interest in recent years due to their exceptional ductility and fracture toughness [9]. Nevertheless, the disadvantage of low yield strength exists in FCC HEAs (typically, 200 MPa in the as-cast state), which significantly limits the potential applications [10]. For example, in the case of track materials for high-speed railroads, it is important to ensure that the track material has high strength and ductility, which is used to resist and cushion the forces, reducing risks and ensuring safety [11]. Increasing the yield strength of HEAs has become a key factor currently. The incorporation of extensive grain boundaries and high dislocation density typically constitutes an effective strategy to impede dislocation motion, thereby markedly augmenting the strength of metallic alloys [12,13]. Moreover, the implementation of precipitation hardening and solid solution strengthening serves as an efficacious method for enhancing material strength. For example, Zhang et al. [14] found that the yield strength and ductility was 512 MPa and 22.1%, respectively, when doping 0.9at% C in CoCrFeNi HEAs. Similarly, Xie et al. [15] used mechanical alloying (MA) and vacuum hot press sintering (VHPS) processes to add 0.1at% N in CoCrFeNiMn HEA and then observed σ phase, Cr_2_N phase, and trace amounts of Cr_23_C_6_ carbides. Meanwhile, the yield strength was increased to 203 MPa compared to the CoCrFeNiMn alloys prepared by the same method, but the ductility was decreased. All these methodologies inherently constrain the proliferation and accumulation of dislocations, which further reduces the degree of strain-hardening and hence the ductility of the alloy. Therefore, increasing strength by conventional methods always leads to a decrease in ductility and is defined as a strength–ductility trade–off.

It has been found that the mechanical properties of the alloys can also be modified by adjusting the processing methods. Choi et al. [16] obtained a fully recrystallized microstructure with a yield strength of 550 MPa and a ductility of 50% by annealing at 600 °C for 1 h after cold rolling a 90% Fe_50_(CoCrMnNi)_50_ alloy. Ji et al. [17] similarly obtained a fully recrystallized microstructure by annealing at 900 °C for 2 h after cold rolling 90% FeMnCoCrNi HEAs. The yield strength and ductility were 644 MPa and 44.1%, respectively. These methods resulted in a fully recrystallized microstructure of the alloy; although the ductility was improved, the strength was not increased. Some research has shown that partial recrystallization annealing after cold rolling was designed to produce heterogeneous grain structure (HGS). This heterogeneous microstructure with different grain sizes can bring good strength–ductility synergy to high-entropy alloys. For example, Miao et al. [18] obtained a yield strength of 848.4 MPa and a ductility of 19.8% by applying a heterogeneous deformation-induced (HDI) plasticity strategy, with (CoCrFeNi)_94_Ti_2_Al_4_ HEAs annealed at 800 °C for 1 h after 80% deep cold rolling. Compared with the usual grain microstructure, HGS can significantly increase the yield strength while amplifying the heterogeneous microstructure to induce strain-hardening and improve the ductility [19,20,21].

In recent years, the concept of high-entropy alloys has been extended to include non-equimolar multicomponent metastable alloys, where deformation mechanisms such as deformation twins and martensitic phase transformation are activated by modulating the phase stability of the alloys, resulting in excellent ductility. However, most of the metastable alloys have high ductility but still low strength. For example, Li et al. [22] prepared a transformation-induced plasticity (TRIP) type Fe_80−x_Mn_x_Co_10_Cr_10_ metastable HEAs with a dual phase microstructure (FCC+HCP). The fully recrystallized microstructure was obtained via cold rolling and annealing, and a yield strength of 330 MPa and a ductility of 70% were obtained. Similarly, Jain et al. [23] prepared fully recrystallized microstructure-metastable Fe_49.8_Mn_30_Co_10_Cr_10_Si_0.2_ HEAs, which obtained a yield strength of 304 MPa while maintaining a ductility of 87%. Therefore, how to further improve the strength while maintaining the excellent ductility of metastable HEAs has become a hot research topic.

In this paper, we prepared the heterogeneous microstructure of Fe_50_Mn_30_Co_10_Cr_10_ metastable HEAs through cold rolling and partial recrystallization annealing treatment. The effects of the annealing temperature on the heterogeneous microstructure and mechanical properties were investigated. The microstructural transformation, strengthening mechanism, and deformation mechanism of the heterogeneous HEAs during the deformation process were analyzed. It is designed to achieve an excellent strength–ductility trade-off by activating multiple deformation mechanisms. We hope that this can provide a reference for the design of high-strength metastable heterogeneous micro-structured HEAs.

## 2. Materials and Methods

The Fe_50_Mn_30_Co_10_Cr_10_(at%) metastable HEAs with 99.9% purity of the constituent elements were prepared via vacuum levitation melting and solidified in a crucible after repeated melting under argon protection (5 times). The alloy ingot was cut into 40 × 15 × 10 mm^3^ blocks via electrical discharge machining, and after grinding and cleaning, the samples were cold rolled from an initial thickness of about 5 mm to a final thickness of about 2.75 mm at room temperature, using multiple passes with a thickness reduction of about 0.05 mm per pass. After that, the alloys were annealed at 600 °C, 650 °C, and 700 °C for 10 min and then quenched in water; henceforth, they are referred to as A600, A650, and A700. The oxide layer and macroscopic defects of the sample were removed via mechanical grinding.

The phase structure of each sample was tested using an X-ray diffractometer (XRD, D8 Advance, Bruker, Karlsruhe, Germany) with a tube voltage of 40 kV and a tube current of 40 mA. The X-ray wavelength was 1.5406 Å, with a scanning range of 20~80°. The scanning rate was 6°/min, and the scanning step size was 0.02°. The lattice parameters were obtained by fitting the XRD peaks on MDI Jade 6 software. The microstructure of the alloy was characterized via field emission scanning electron microscopy (SEM, Thermo Scientific Apreo 2C, 20 KV, America), an electron backscatter diffraction system (EBSD, EDAX Velocity Super, 20 KV, America) with a step size of 0.15 μm, and transmission electron microscopy (TEM, FEI Talos F200X, 200 KV, America). XRD and EBSD samples were prepared through electropolishing in ethanol (77%)–perchloric acid (23%) solution. TEM samples were mechanically ground to a thickness of 50 μm or less, and then a 3 mm disk was punched out from these samples, which was then thinned to electron transparency using the Gatan 695 Precision Ion Polishing System (PIPS). Microvickers hardness tester (THV-10D, Beijing Era United Technology Co., Ltd, Beijing, China) was utilized for the determination of material hardness. The test load was 1N. Wach sample was measured six times, the maximum and minimum values were discarded, and the average was computed to obtain the final hardness. The dog bone-shaped specimens for the tensile tests, with a gauge dimension of 40 × 8 × 1 mm^3^, were cut from the alloy in the rolling direction (RD) using electrical discharge machining. Tensile testing was conducted on the Instron Legend 2382 machine at a strain rate of 10^−3^/s. For each alloy, three samples underwent tensile testing to ensure repeatability. Among them, the yield strength (YS) is calculated as $\sigma_{\mathrm{YS}}=F/A$, where F is the stress corresponding to the yield stage and A is the original cross-sectional area of the material. The ultimate tensile strength (UTS) is calculated as $\sigma_{\mathrm{UTS}}=F_{b}/A$, where F_b_ is the maximum stress that the material can endure during the tensile test and A is the original cross-sectional area of the material. The fracture elongation (FE) is calculated as $\delta =((L_{1}-L_{0})/L_{0})\times 100\%$, where L_0_ is the original scale length of the material before the tensile test and L_1_ is the scale length of the material after tensile fracture.

## 3. Results

### 3.1. Microstructure After Annealing

**Figure 1:**
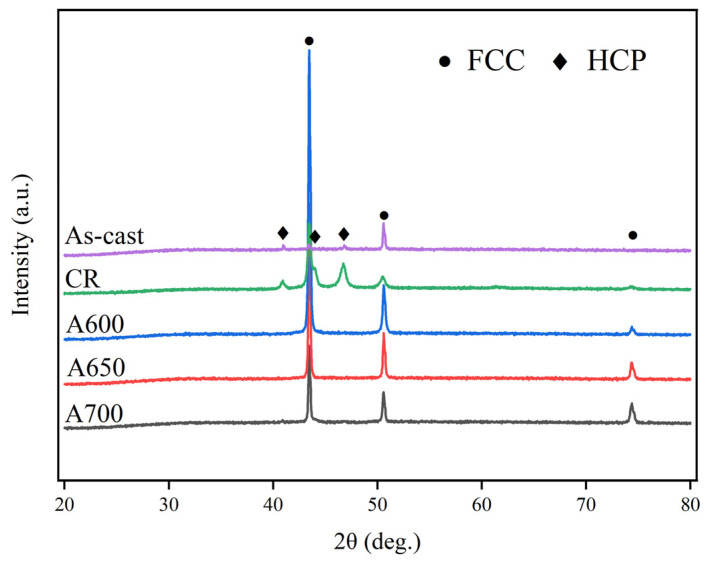
XRD diffraction patterns at different samples.

**Figure 2:**
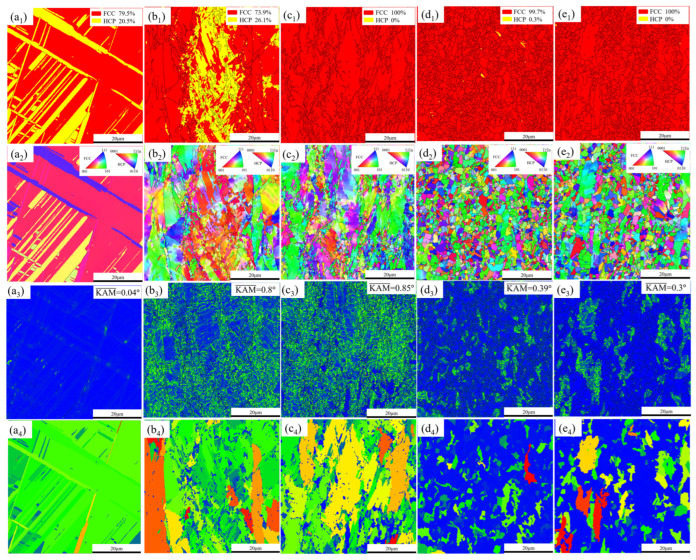
EBSD maps of the (**a**) as-cast alloy, (**b**) CR alloy, (**c**) A600 alloy, (**d**) A650 alloy, and (**e**) A700 alloy. (**a_1_**–**e_1_**) phase distribution diagrams; (**a_2_**–**e_2_**) IPF diagrams; (**a_3_**–**e_3_**) KAM maps; (**a_4_**–**e_4_**) GOS maps.

The XRD patterns of the as-cast, cold-rolled (CR), A600, A650, and A700 samples are shown in Figure 1. Both the as-cast and CR samples showed FCC and HCP peaks, while the annealed samples (A600, A650, and A700) had a single FCC peak without an HCP peak. The EBSD patterns of as-cast, CR, A600, A650, and A700 samples are shown in Figure 2. The microstructure consisted of recrystallized fine grains (RFG, blue area in Figure 2c_4_–e_4_) and residual deformed grains (RDG). This combination of RFG and RDG was called the heterogeneous microstructure. The phase distribution diagram (Figure 2a_1_–e_1_) shows the phase structure of the as-cast, CR, and annealed samples (A600, A650, and A700), which was consistent with the XRD results (Figure 1). As shown in Figure 2b_1_, the sample underwent an FCC → HCP transformation during deformation, resulting in 26.1% HCP content. Then, after annealing treatment, the HCP content decreased, and the annealed samples had a single FCC phase structure, which indicates that the HCP → FCC reverse phase transformation occurred during annealing. The IPF diagram (Figure 2b_2_–e_2_) showed that the grains within the alloy exhibit no distinct preferred orientation, suggesting that the annealing temperature exerted negligible influence on the grain orientation of the alloys. The average grain sizes (statistically obtained by Aztec Crystal software, version 2.1) of these five samples were calculated to be 28.03 ± 5.12 μm, 0.71 ± 0.18 μm, 0.79 ± 0.3 μm, 1.10 ± 0.57 μm, and 1.16 ± 0.6 μm, respectively. The KAM map represented the average misorientation angle within the grain, which reflected the distribution of dislocation and strain. In Figure 2c_3_, it can be seen that the A600 sample had a high density of dislocations in larger regions. The blue region signified a low KAM value, with the grain boundaries in these regions predominantly being high-angle grain boundaries (HAGB). The green regions are composed of deformed grains with high KAM values. In these regions, the majority of the grain boundaries were characterized as low-angle grain boundaries (LAGB). The grain orientation spread (GOS) maps in Figure 2c_4_–e_4_ show the degree of recrystallization for three samples. The range used in the GOS analysis was from 0 to 20. The blue areas represent the recrystallization zone. Statistically, the recrystallized zone fractions of the A600, A650, and A700 samples were 10.2%, 65.8%, and 74.7%, respectively.

**Figure 3:**
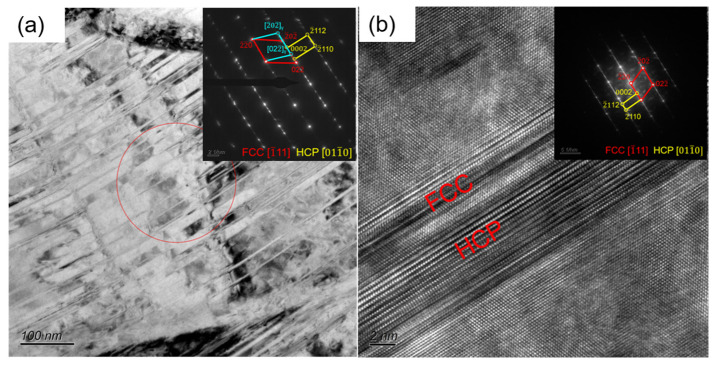
TEM image of CR sample: (**a**) BF image for deformed twins and HCP laths with the SAED inset; (**b**) HR TEM image for deformed HCP lath.

**Figure 4:**
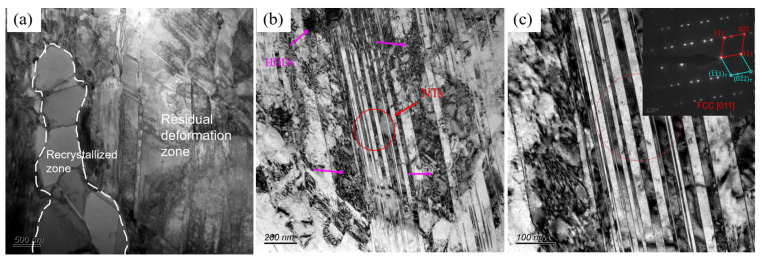
TEM image of A600 sample: (**a**) BF image of heterogeneous structure; (**b**) BF image for annealing twins; (**c**) the SAED pattern of the red region in (**b**).

The microstructural TEM image of the CR sample is shown in Figure 3, from which it is evident that the twins and HCP, which were closely associated, were analyzed as deformation twins, and stress–induced FCC → HCP phase transformation. In order to further clarify the effect of annealing temperature on the microstructure, the A600 sample was observed by TEM, as shown in Figure 4a, with a typical heterogeneous structure which contained recrystallized and residual deformation zones. In the residual deformation zones, there existed nano-scale twins (NTs) and a high density of dislocations (HDDs). Numerous parallel lamellae can be observed in the BF TEM image (Figure 4b). Figure 4c shows an enlarged red circle in b. The SAED pattern at the upper right corner shows that these slabs are twins. This is also consistent with the previous EBSD (Figure 2) results.

### 3.2. Mechanical Properties

**Figure 5:**
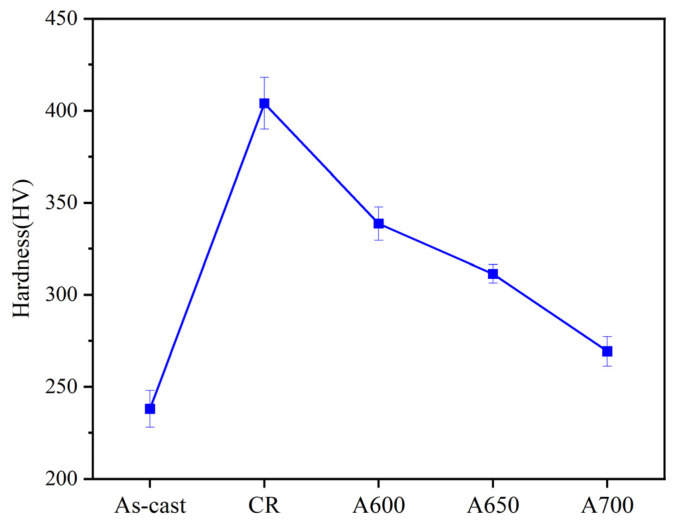
Microhardness of as-cast, CR, and annealed samples (A600, A650, and A700).

**Figure 6:**
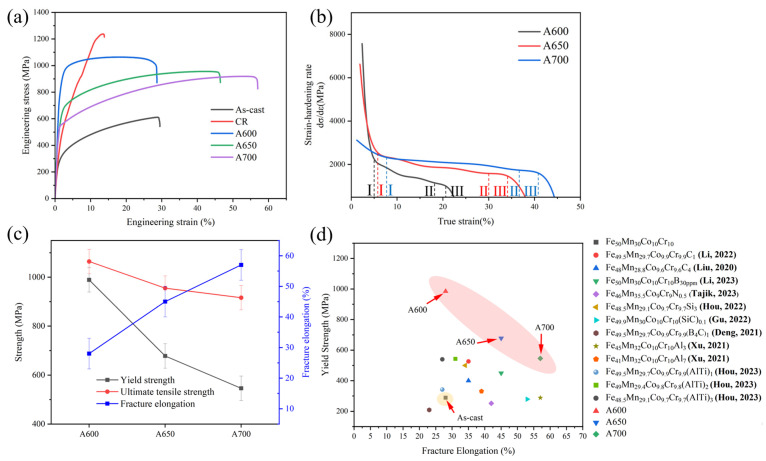
Tensile properties of the alloys: (**a**) engineering stress–strain curves; (**b**) strain-hardening rate curves for alloys A600, A650, and A700; (**c**) the variation of yield strength, ultimate tensile strength, and fracture elongation with annealing temperature; (**d**) a comparison of the tensile properties of the present study with those of other alloys of the same composition [24,25,26,27,28,29,30,31,32].

Figure 5 shows the micro-hardness of Fe_50_Mn_30_Co_10_Cr_10_ high-entropy alloys at different annealing temperatures. It can be seen that the microhardness of the alloy decreases with the increasing annealing temperature, which is closely related to the microstructural changes induced by annealing. The increase in recrystallized grains possessing lower hardness, coupled with the reduction in residual deformed grains characterized by higher hardness, collectively results in the degradation of the alloy’s hardness. Figure 6a shows the engineering stress–strain curves for the A600, A650, and A700 samples. The specific tensile properties are summarized in Table 1. Compared to the as-cast sample, the annealed samples showed a significant increase in strength and ductility. The yield strengths of the A600, A650, and A700 samples were 989 MPa, 678 MPa, and 546 MPa, respectively, while the ductility was 28%, 45%, and 57%, respectively. The strain-hardening curves of these three samples are shown in Figure 6b, and they exhibit a three-stage strain-hardening behavior. First, the rate of strain-hardening underwent a precipitous decline with the increase in the true strain (Stage I). Subsequently, the strain-hardening rate decreased more slowly (Stage II). Finally, as the true strain continued to increase, the strain-hardening rate decreased rapidly until the specimen broke (Stage III). It was apparent that the strain-hardening capacity was intimately associated with the annealing temperature of the alloy; that is, the strain-hardening rate increased in correlation with the ascending annealing temperature at a consistent applied strain. Figure 6c shows the effects of annealing temperature on the tensile properties of the alloy. As the annealing temperature increases, the strength of the alloy diminishes, while conversely, the elongation tends to increase. Figure 6d shows the YS versus FE of Fe_50_Mn_30_Co_10_Cr_10_ HEA compared to other alloys with the same or similar composition; all of them were analyzed by engineering stress–strain curves, and the red area showed the results of this study. The strength and ductility of alloys with heterogeneous microstructures was significantly better than those of the as-cast sample and other works. Obviously, it is indicated that the A600 alloy possesses superior tensile properties. Therefore, the preparation of HEAs via heterogeneous structure treatment gave it excellent tensile properties and provided a significant reference for the advancement of other HEA in practical applications.

### 3.3. Microstructure After Deformation

**Figure 7:**
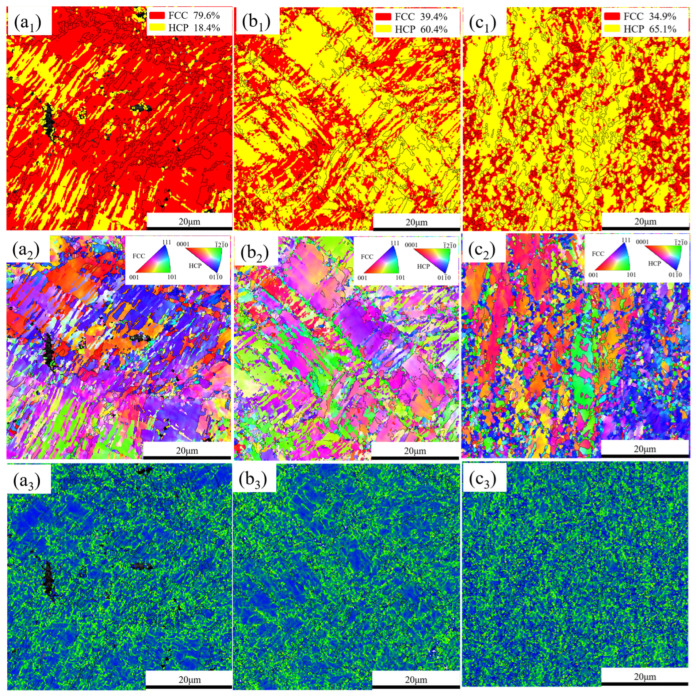
EBSD maps for different deformations: (**a**) 15%, (**b**) 30%, and (**c**) fracture. (**a_1_**–**c_1_**) Phase distribution diagrams; (**a_2_**–**c_2_**) IPF diagrams; (**a_3_**–**c_3_**) KAM maps.

To examine the influence of deformation on the content of HCP, EBSD analysis was carried out on the A600 sample after different strain (15%, 30%, and fracture). As shown in Figure 7a_1_–c_1_, the HCP content increased from 18.4% to 65.1% as the strain increased from 15% to fracture. Therefore, stress–induced FCC → HCP phase transformation has emerged as the predominant deformation mechanism of the current alloy. The IPF diagram (Figure 7a_2_–c_2_) shows that the grains within the alloy exhibited no distinct preferred orientation, suggesting that the deformation exerted a negligible effect on the grain orientation of the alloys. The KAM maps (Figure 7a_3_–c_3_) demonstrated a fairly homogeneous distribution of local strain without indications of localized strain in proximity to the HCP phase. Meanwhile, it can be seen that the area of magnitude of the local misorientation intensifies with the increment of strain.

**Figure 8:**
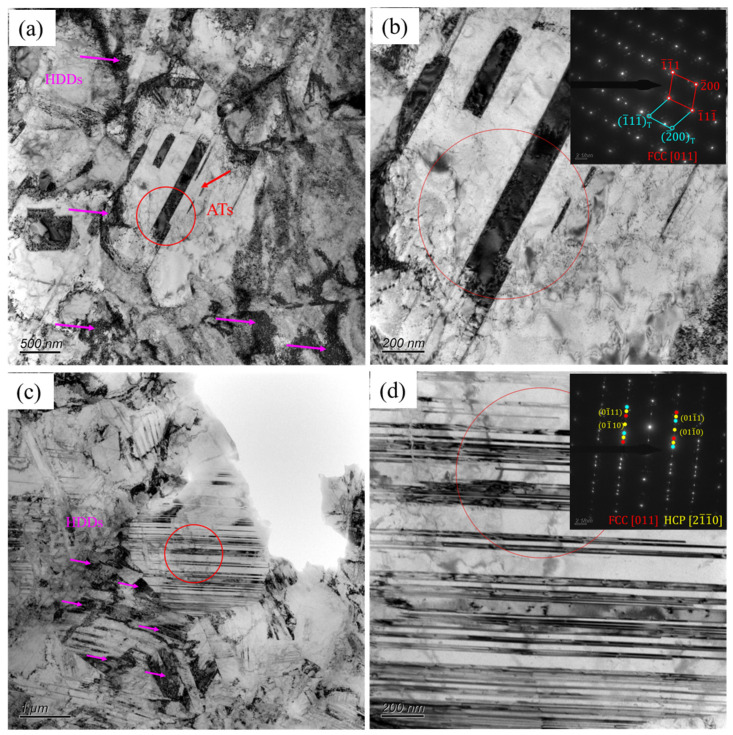
TEM images of A600 alloy after 15% deformation: (**a**,**c**) BF images; (**b**) BF image for annealing twins with the SAED inset; (**d**) BF image for deformed HCP laths with the SAED inset.

To further explore the deformation microstructure of the A600 sample to reveal its deformation mechanism, TEM characterization tests were conducted. When the strain was 15%, the strain-induced FCC → HCP transformation emerged as the predominant deformation mechanism. Figure 8a,c shows the deformation microstructure of A600 sample when the tensile strain was 15%. Some high density of dislocations (HDDs) and annealing twins (ATs) were observed. As well as the FCC matrix, the selected area electron diffraction (SAED) patterns also revealed distinct HCP diffraction spots. Figure 8b shows an enlarged view of the red-circled portion of Figure 8a and the corresponding SAED diagram, from which a more obvious twin pattern about the [011]_γ_ plane can be seen. Figure 8d features an enlarged perspective of the red-circled portion of Figure 8c and the corresponding SAED plot, from which the twin pattern, as well as the HCP spots, can be seen.

**Figure 9:**
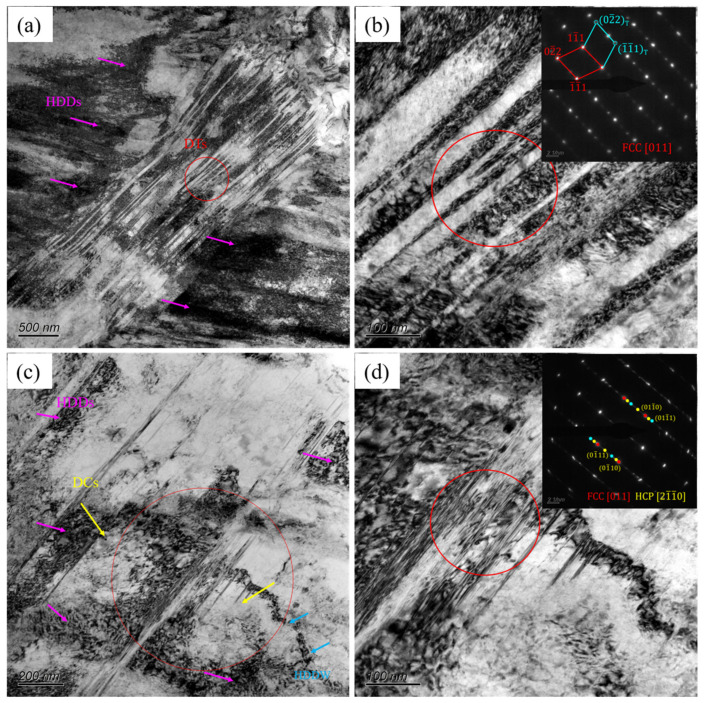
TEM images of A600 alloy after 30% deformation: (**a**,**c**) BF images; (**b**) BF image for deformed twins with the SAED inset; (**d**) BF image for deformed HCP laths with the SAED inset.

Figure 9a is a BF image of A600 alloy with 30% deformation, showing that a profusion of dislocations intertwines to create a high density of dislocations and deformation twins (DTs) during tensile deformation. The SAED pattern corresponds to the diffraction of the region along the [011]_γ_ axis in Figure 9b, confirming the existence of layered DTs. At the same time, some dislocations slip to form dislocation cells (DCs) in Figure 9c, and dislocation cell-form deformations form randomly distributed high-density dislocation walls (HDDWs) [33]. The development of DTs and HCP phase introduced interfaces, which refined the microstructure and diminished the average free path of dislocation slip during tensile deformation, thereby imparting a dynamic Hall–Petch hardening for the alloy [34]. The BF image in Figure 9d illustrate the coexistence of HCP laths and some DTs. At the same time, it can be seen that the dislocation distribution was mixed between HCP, which indicates the HCP hindered the dislocation movement. A considerable multitude of FCC → HCP transformation led to the increase in HCP content (18.4% → 60.4%; Figure 7a_1_,b_1_), and the TRIP effect was significant. The SAED pattern corresponded to the diffraction of the region along the [011]_γ_ and [2$\bar{1}\bar{1}0$]_ɛ_ axes.

**Figure 10:**
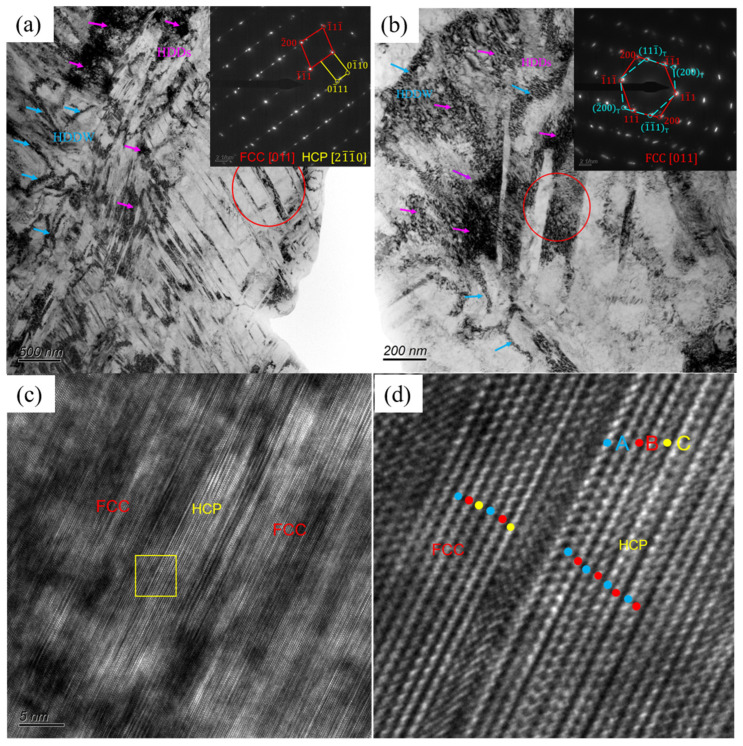
TEM images of A600 alloy after fracture: (**a**) BF image for deformed HCP laths with the SAED inset; (**b**) BF image for deformed twins with the SAED inset; (**c**) HR TEM image for deformed HCP lath; (**d**) the FFT filtered image corresponding to the yellow box area in (**c**).

Similarly, Figure 10a,b shows the bright field (BF) images of the A600 alloy after fracture. It can be seen that the dislocations were tangled with each other to form more HDDs and HDDWs, and the inset showed the SAED pattern in the red-circle region, confirming the presence of the DTs and HCP phases. Further HR TEM and FFT filtering analysis (Figure 10c,d) shows that the interface between the FCC and HCP phases was intricately interrelated, and the atomic stacking sequence at the interface of the two phases ranged from …ABCABC… (FCC phase) to …ABABAB… (HCP phase), echoing the findings reported in previous studies [35].

## 4. Discussion

### 4.1. Strengthening Mechanism

The degree of dislocation motion obstruction determines the strength of the alloys. In homogeneous materials, the enhancement of strength can be attributed to conventional strengthening mechanisms, including solid solution strengthening, precipitation hardening, dislocation strengthening, and grain boundary strengthening [36]. However, there is an additional mechanism in heterostructured (HS) materials, namely, heterogeneous deformation-induced (HDI) strengthening, which helps achieve a synergy between strength and ductility. In this paper, the heterogeneous microstructure of Fe_50_Mn_30_Co_10_Cr_10_ HEAs was prepared via cold rolling and partial recrystallization annealing, and the yield strength was increased by 689 MPa compared with the as-cast sample (Figure 6a). This phenomenon was a result of the synergistic action of various strengthening mechanisms, including dislocation strengthening ($\sigma_{D}$), grain boundary strengthening ($\sigma_{G}$), twin ($\sigma_{T}$)-induced strengthening, and HDI strengthening ($\sigma_{HDI}$). Therefore, the yield strength of the A600 sample can be expressed as Formula (1).

(1) $$\sigma =\sigma_{D}+\sigma_{G}+\sigma_{T}+\sigma_{HDI}$$

Dislocation strengthening ($\sigma_{D}$) can be obtained vis Formula (2). Here, M is the Taylor factor (3.06); α is a constant with a value of 0.2 [37]; G is the modulus of rigidity (G = 76 GPa) [38]; b is the Burgers vector ($\sqrt{2}/2\times a$ (lattice parameter, which can be determined by fitting the XRD data)); ρ is the dislocation density, which can be calculated cia Formula (3); θ denotes the misorientation angle, which is assessed by the average KAM value obtained from EBSD analysis; and μ represents the scanning step size of 0.15 μm.

(2) $$\sigma_{D}=\mathrm{MG}\alpha b\sqrt{\rho }$$

(3) ρ=2θ/μb

The contribution of grain boundary strengthening ($\sigma_{G}$) can be determined through the application of Hall–Petch Equation (4). Here, d is the average grain size of the recrystallization region (0.79 μm); and k is the Hall–Petch coefficient (226 MPa/$\mu m^{-0.5}$ [39]).

(4) $$\sigma_{G}=kd^{-0.5}$$

**Figure 11:**
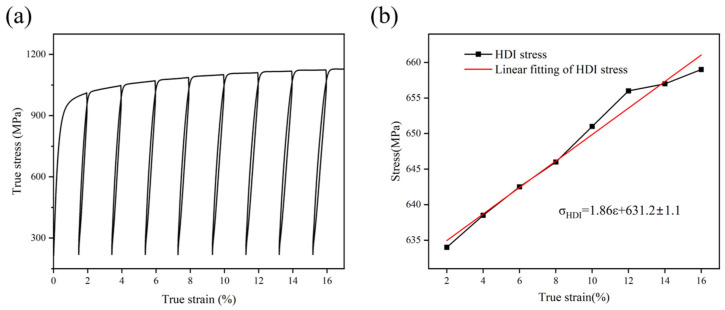
(**a**) Load–unload cycle (LUR) curves for A600 alloy. (**b**) Back stress hardening.

To elucidate the contribution of HDI strengthening to strength, a unidirectional quasi-static cyclic loading and unloading experiment was performed on the first 16% strain range of the A600 sample, as shown in Figure 11. It was found that $\sigma_{HDI}$ could be obtained from the loading and unloading curve, and the calculation in Formula (5) follows below. Here, $\sigma_{u}$ is the unloading yield point stress; and $\sigma_{r}$ is the loading yield point stress. By fitting the first 16% approximately linear back stress, its contribution to strength is 631.2 MPa.

(5) $$\sigma_{HDI}=(\sigma_{u}+\sigma_{r})/2$$

The formation of annealing twins is primarily ascribed to the migration of recrystallized grain boundaries, while the slow diffusion effect in HEA hinders the migration rate of grain boundaries and also makes it more susceptible to the formation of annealing twins. Furthermore, the annealing twin boundaries within the recrystallization region and the deformation twin boundaries in the deformation region can be used as impediments to dislocation slip. The strengthening effect of twin boundaries is quantified by offsetting their contribution from other strength components.

In summary, it was found that $\sigma_{HDI}$ was the main factor responsible for the higher yield strength of the A600 sample compared to the as-cast sample. In other words, partial recrystallization annealing allowed the alloy to acquire a microstructure with a residual deformation zone along with a fine-grained recrystallized zone [40,41], which would greatly improve the strength of the alloys.

### 4.2. Deformation Mechanism

The ductility of an alloy is contingent upon its deformation mechanisms, including dislocation slip, twinning, and phase transformation [42]. Based on the previous discussion, the HCP phase volume fraction of the A600 sample increased significantly after tensile fracture (Figure 7c_1_). The continuous stress–induced FCC → HCP phase transformation during deformation was congruent with the dynamic phase transformation behavior of Fe_50_Mn_30_Co_10_Cr_10_ high-entropy alloys [22]. The continuous phase transformation not only dissipates strain storage energy to alleviate stress accumulation; it also results in an augmentation of the phase boundary, which provides an additional barrier for dislocation slip, thus fostering strain-hardening through the dynamic Hall–Petch effect and giving rise to a TRIP effect [22].

During the initial phase of deformation, as full and partial dislocations undergo slip, the dislocation density in the FCC matrix increased rapidly, forming a high-density dislocation region (Figure 7a_3_). The higher the dislocation density in the FCC phase region, the higher the plastic strain. Therefore, the dislocation slip and the TRIP effect were the main deformation mechanisms in the initial stage of plastic deformation.

With a further increase in deformation, the stress induced FCC → HCP phase transformation, which was reflected by a significant increase in the HCP phase fraction (Figure 7b_1_). Usually, twin nucleation occurs in grains with lower dislocation density because the twinning mechanism exceeds the dislocation slip mechanism in controlling the deformation behavior [43]. However, deformation twins could arise within grains with high dislocation density (Figure 9a) in HEA, which activated the TWIP effect [44] and improved their strength and ductility [45]. At the same time, due to the strain incompatibility between the recrystallization zone and the residual deformation zone, geometrically necessary dislocations (GNDs) were generated to ensure the continuity of strain across grain boundaries (GB) [20,46]. Also, GNDs can generate long-range internal stresses—that is, back stresses—in the recrystallization zone to counteract the applied shear stresses [47,48]. Moreover, dislocations build up at the grain boundaries, causing stress concentration and generating forward stresses in the residual deformation zone [48]. The heterogeneous deformation-induced (HDI) hardening effect caused by the interaction of back and forward stresses produces additional strain-hardening in HS materials [48,49]. According to Hart theory and Considère standard, the higher degree of strain-hardening helps to delay the occurrence of necking during plastic deformation and improve ductility, which was also consistent with what is shown in Figure 6b. Therefore, HDI strain-hardening was the main characteristic in the middle stage of deformation. In the late stage of deformation, the stress–induced FCC → HCP phase transformation tended to saturate, and the interaction between dislocations and martensite also promoted HDI strain-hardening (Figure 11b), which, when combined with the TRIP effect, enabled the A600 sample to acquire an excellent strength–ductility balance.

Overall, the plastic deformation of the alloy encompasses a spectrum of structural features. It is the interplay among these diverse structural characteristics that enables the alloy under investigation to exhibit high yield strength and excellent ductility at the same time. This process of creating a heterogeneous microstructure via cold rolling and partial recrystallization annealing with the aim of enhancing the mechanical properties of the alloy is designed to provide an efficacious approach for the future strengthening and toughening of the alloy.

## 5. Conclusions

In this work, metastable high-entropy alloys (Fe_50_Mn_30_Co_10_Cr_10_) with a heterogeneous microstructure were prepared via cold-rolling and subsequent partial recrystallization annealing treatment. Compared with the as-cast samples, the heterogeneous microstructure (A600, A650, and A700) samples exhibited an excellent strength–ductility synergy. By employing a suite of characterization techniques, the impact of microstructure attributes on mechanical properties was elucidated, and the pertinent strengthening and deformation mechanisms were delineated. The main results are concluded as follows:

1. In the cold rolling process, in addition to some deformed FCC grains, there were some grains which occured the stress–induced FCC → HCP phase transformation. Subsequently, in the annealing process, some grains induced the HCP → FCC reverse phase transformation, and the remaining grains formed a heterogeneous microstructure characterized via the formation of deformed FCC grains with dense dislocations and the recrystallization of fine FCC grains.
2. The combined effect of multiple strengthening mechanisms, including dislocation strengthening, grain boundary strengthening, and heterogeneous deformation-induced strengthening, resulted in a high yield strength of 989 MPa for the A600 sample with a heterogeneous microstructure.
3. The TWIP effect activated by the deformation twins could continuously provide a source of strain-hardening and improve ductility while maintaining high strength. At the same time, the TRIP effect activated by stress induced FCC → HCP phase transformation combined with heterogeneous deformation-induced strain-hardening further improved the ductility. The combined effect of multiple deformation mechanisms realized a good combination of strength and ductility.

The above results provide a reference for improving the strength and ductility of metastable high-entropy alloys. In the future research, we believe that the adjustment of alloy composition should be carried out to further improve the strength and ductility of the alloys by precipitation strengthening, grain refinement, and other means, combined with heterogeneous microstructure, while maintaining the metastable state. Meanwhile, the low-temperature mechanical properties can be studied to provide reference for other low-temperature applications.

## Figures and Tables

**Table 1:** The specific numerical tensile properties of each alloy.

Sample Name	YS (MPa)	UTS (MPa)	FE (%)
As-cast	300 ± 5	610 ± 7	29 ± 2
CR	1214 ± 5	1237 ± 7	13 ± 1
A600	989 ± 8	1064 ± 11	28 ± 3
A650	678 ± 9	955 ± 12	45 ± 2
A700	546 ± 7	916 ± 9	57 ± 3

## Data Availability

The raw data supporting the conclusions of this article will be made available by the authors upon request.
